# Acting on the Voicelessness Challenge to Democratic Deliberation: A Six‐Actor Framework

**DOI:** 10.1111/bioe.70180

**Published:** 2026-09-09

**Authors:** Julian C. Jamison

**Affiliations:** ^1^ University of Exeter Exeter Devon UK

## Abstract

Political philosophers have long recognized that key stakeholders in policy decisions—children, future generations, nonhuman animals, and individuals with severe cognitive impairments—are structurally unable to participate in the deliberative processes meant to legitimate those decisions. This paper argues that such “voicelessness” is not a marginal complication to be accommodated at the edges of deliberative democracy (DD), but a first‐order problem for exactly the large‐scale, high‐stakes domains—health, biotechnology, and environmental policy chief among them—where the voiceless often constitute the majority of those affected. This exclusion risks systematically neglecting crucial interests and suggests that some degree of surrogacy or representation by proxy is functionally unavoidable, whether or not DD's more demanding commitment to reasoned, preference‐transforming deliberation is in play. Building on this recognition, and after critiquing the feasibility of idealized deliberative democracy as well as partial solutions implied by existing work, this paper's central contribution is a more pragmatic, ethically robust Six‐Actor Framework. The framework delineates specific roles for policymakers, target populations, general laypeople, technical domain experts, and two novel categories: “benevolent partisans” (e.g., caregivers) and “agent‐specific experts” tasked with interpreting or forecasting the interests of the voiceless. The utility of this structured approach is illustrated through three high‐stakes policy domains: constructing global health metrics (DALYs) for cost‐effectiveness analysis, animal welfare regulation, and the challenges involved when impacting the very existence of future people. This paper offers a path toward a policy process that is both practicable and ethically defensible, specifically for large‐scale, high‐priority global challenges.

## 1 Introduction

Imagine a policymaker G who must choose between two equally costly programs: intervention A increases the consumption of a given population X by 10% for the remainder of their lives, but doesn't change their health or lifespan; intervention B increases the lifespan of the same population by 5 years (say via a constant decrease in age‐specific mortality rates), but doesn't change their consumption levels. Which program should G choose? What process should G use to arrive at their decision?

One natural option is to ask population X for their preference and implement the majority choice. In some scenarios, this appeals to fairness, procedural justice, and efficiency: if agents know their own preferences better than outsiders do, this aggregates information toward the best outcome. However, there are conceptual reasons to doubt the universal optimality of this approach. Individuals are often poor at “affective forecasting”—predicting their own future wellbeing as a function of changed circumstances—even when fully informed and free of any political bias.^1^ Separately, and even where individual self‐knowledge is accurate, critics like Jason Brennan [3] have argued that the quality of input from a general electorate can be hampered by systematic biases or a lack of specialized knowledge.

This article focuses on a more fundamental barrier: the practical incapacity for participation faced by many relevant stakeholders. Political philosophers have long observed this problem for particular groups—Feinberg [4] first grouped nonhuman animals, future generations, and young children together as entities that cannot claim rights or press their own interests through ordinary participatory channels, and subsequent work has developed this insight for children specifically [5]. This paper's aim is not to rediscover this observation, but to show that it is a first‐order design problem for exactly the domains—health, biotechnology, environmental policy—where the voiceless are not a marginal constituency but often the majority of those affected, and to supply an operational framework for responding to it.

To ground this discussion, I define deliberative democracy (DD) as a process of “reasoned engagement” with the public,^2^ underpinned by two distinct rationales: an epistemic rationale, under which diverse input improves the fidelity of the information available for decision‐making, and a process rationale, under which participation itself—and specifically the exchange of reasons that can transform, rather than merely register, participants' preferences—confers legitimacy. Voicelessness is most obviously fatal to the process rationale: an agent who cannot argue cannot have her preferences transformed by argument, nor can she transform anyone else's. But the problem is not thereby resolved by retreating to a purely epistemic or preference‐aggregating conception of DD. Even under such weaker versions—closer to simple preference elicitation or an aggregation of independent judgments—the voiceless remain excluded, since no aggregation mechanism, however well designed, can register information from an agent incapable of producing a signal in the first place.

I define voicelessness as this practical incapacity for engagement, arising from two primary sources:

1. Developmental, Biological, and Cognitive: Young children, nonhuman animals, and individuals with severe cognitive impairments or misperceptions of reality, all of whom share an incapacity for reasoned engagement grounded in cognitive or developmental limitation.
2. Temporal: Future generations, who do not yet exist and so cannot engage regardless of cognitive capacity, and on the same logic one's own sufficiently distant future self, who is not simply a continuous extension of the present self but is in important ways equivalent to a distinct other person.

The primary point is that it is impossible to elicit useful input directly from these groups via standard procedures. This implies that a certain amount of surrogacy is necessary in most important policy decisions. While proponents of DD often view the process as a substitute for aggregating the preferences of *citizens*, I argue that in domains like health and science policy, the focus must shift to the interests of *beneficiaries*—as distinct from those who supply or implement policy and are typically well placed to advocate for themselves—many of whom are inherently silent.

This paper seeks to bridge the gap between political theory and practical policy through three main contributions, building on and operationalizing a problem that political philosophy has identified but left largely unaddressed at the level of policy design:

- Scope and Synthesis: Formally linking the population ethics literature with the literature on deliberative democracy and the “all‐affected principle” [7], to show that voicelessness is a first‐order problem for precisely the large‐scale, high‐priority domains in which the voiceless often constitute the majority of those affected, rather than an isolated exception to be retrofitted onto standard deliberative procedures.
- The Six‐Actor Framework: A novel conceptual taxonomy that identifies distinct roles for “benevolent partisans” and “agent‐specific experts” to represent the voiceless, providing an operational structure that existing political‐theoretic treatments of voicelessness do not supply.
- Empirical and Practical Grounding: Bringing real‐world data from development economics and behavioral science—areas often missing from principle‐level debates—to show where deliberation fails and expert guidance can succeed, and applying a “Who and When” matrix for surrogacy to case studies where this approach would plausibly change the ultimate policy decision.

Section 2 critiques DD via conceptual and empirical constraints on public input. Section 3 proposes the Six‐Actor Framework, offering an explicit defense of how to weight the interests of those who cannot speak. Section 4 illustrates these ideas through three examples: global health metrics, animal welfare legislation, and population ethics. Section 5 concludes with next steps for implementing agent expertise in high‐priority global domains, including a closing discussion of how this same logic of temporal incapacity applies not only to future generations but to one's own sufficiently distant future self.

## 2 Theoretical and Empirical Barriers to Deliberative Inclusion

Deliberative democracy is typically defended on the two grounds introduced in Section 1: an epistemic rationale, that pooling diverse perspectives avoids biased perspectives, group‐think, and abbreviated search; and a process rationale, that participation and argumentative exchange confer legitimacy. Both face real complications even when every affected party is capable of participating. On the epistemic side, Benson [8] shows formally that diversity's epistemic benefits do not by themselves establish that deliberative democracy is superior to other collective‐decision mechanisms, particularly once stakeholders have incentives to misreport their private information. These are genuine problems, but this paper sets them aside to focus on a more fundamental barrier that persists even under the most favorable assumptions about aggregation among fully capable participants.

### 2.1 The Practical Incapacity for Full Inclusion

The defining characteristic of deliberative democracy is reasoned engagement with members of the public. Following the “all‐affected principle,” the relevant public should include all those impacted by a decision. However, key members of this public are practically incapable of such engagement, whether for developmental, biological, or cognitive reasons (very young children, nonhuman animals, individuals with severe cognitive impairments) or for temporal reasons (future generations, who do not yet exist). While older children or adults with more limited cognitive capacity might engage in limited deliberation, the more extreme examples are sufficient to show that full democratic deliberation is practically infeasible for many pertinent decision contexts.

The relevant groups can be represented in various ways, but they cannot represent themselves, which was the original impetus for the project of democratic deliberation. Incorporating only the preferences of groups that can speak introduces a non‐random selection effect. Under an epistemic rationale, this information may be biased; under a process rationale, it leads to increased asymmetry where some groups advocate for themselves while others remain silent. A simple dictum advising deference to “the community” or “the beneficiaries” is insufficient because these groups are importantly heterogeneous, containing key members unable to express themselves.

Is partial deliberative democracy at least better than none? Not necessarily, since there is a non‐random selection effect introduced by incorporating the preferences and experiences of only some groups. Under the epistemic rationale this new information, although valuable in itself, may well be biased (consider the contrasting views between current and future generations) and the net effect could go in either direction. Under the process rationale things look only superficially better: naively, more representation might seem to imply more legitimacy, but as Section 1 argues, DD's process rationale rests specifically on argumentative exchange transforming preferences, not merely registering them—and partial inclusion cannot approximate this for the excluded groups, since there is no argument in which they can take part. The result is increased asymmetry and inequity: under “technocracy” all groups are only indirectly represented, whereas under DD some groups are directly advocating for themselves, and having their views transformed through argument with others, while some other groups are unable to do so at all.^3^

### 2.2 Synthesizing Population Ethics and Political Theory

Political theory has identified voicelessness as a problem for deliberative legitimacy (Section 1), but has generally treated it as a normative diagnosis rather than an operational design question: establishing that a group cannot deliberate does not by itself say how much weight to give their absent interests, or through what institutional channel. Population ethics, meanwhile, has developed rigorous tools for exactly this weighting problem, largely in the abstract and disconnected from institutional design. Connecting the two fields is necessary precisely because addressing voicelessness in practice requires both a normatively defensible way of weighing absent interests and a concrete mechanism for incorporating that weighting into actual policy—which is the project of this paper. The tension this connection reveals is structural: the “all‐affected principle” [7] necessitates a scope of inclusion that the standard deliberative requirement of “reasoned engagement” [6] practically precludes.

Democratic theory generally faces a foundational problem of boundaries: who constitutes the *demos*? Goodin [7] treats this as among the most basic unresolved problems for democratic legitimacy as such, and Arrhenius [9] notes that a decision isn't “democratic” simply because a group deliberates; it must be the *right* group. DD inherits this problem and compounds it: because DD's process rationale depends on transformative argumentative exchange rather than mere aggregation, drawing the boundary wrong does not just misallocate votes, it forecloses any possibility of argument with those excluded. This “boundary problem” was originally treated as a spatial or jurisdictional issue, but Thompson [10] extended it to the temporal dimension over a decade before it became a prominent theme in the literature, analyzing how democratic institutions structurally privilege present preferences over future ones; Meyer [11] develops this critique further.

Rawls [12] provides an early, if unintentional, framework for intergenerational deliberation through the “Original Position” and the Just Savings Principle (JSP): behind a hypothetical veil of ignorance, not knowing to which generation they belong, parties agree on how much each generation should save for the next, on the assumption that all generations follow the same rule. Rawls is explicit that this cannot describe an actual exchange between generations^4^; the JSP therefore functions as a *pre‐commitment mechanism*, binding current deliberation to a hypothetical, not an actual, intergenerational consensus—which is telling for this paper's argument: even the most influential attempt to bring future generations into deliberative theory abandons real deliberation with them for a one‐sided thought experiment. DD, by contrast, is generally understood to require *actual* reasoned exchange among *present* parties [6]; the JSP's reliance on a hypothetical device is not a minor technicality but an implicit concession that real deliberation with the voiceless is unavailable.

An important claim in this paper is that DD often prioritizes the *process* of talk over the *fidelity* of the result, and that this prioritization becomes untenable once some of those affected cannot talk at all. Gosseries [14] makes a related point about future generations specifically, describing them as both “voiceless” (unable to articulate preferences) and “toothless” (unable to hold any purported representative accountable, since they can neither authorize nor later sanction a proxy). Both terms describe the same procedural relationship from opposite ends—input and enforcement—rather than a split between procedural and substantive concerns. Gosseries's conclusion is correspondingly pessimistic: because future generations can neither authorize nor discipline a representative, he argues that policy toward them is best described not as “represented” but as merely “a‐legitimate”—neither legitimate nor illegitimate, since the concept of democratic legitimacy does not have purchase in the first place. This is a stronger version of the difficulty DD faces here: it is not just that the voiceless lack a channel for input, but that even a well‐intentioned proxy relationship on their behalf cannot be held accountable by the party it claims to serve.

The population ethics literature also remains (perhaps purposefully) vague on a key “base‐year” problem: should we represent future generations using our current values (applied to their predicted lives) or their likely future values? MacAskill [15] highlights the risk of “value lock‐in,” where current deliberation cements a moral framework that future agents might find abhorrent. This is a classic principal‐agent problem with a temporal twist. If we assume future values will change, we face a choice: do we deliberate on behalf of their *actual* future selves (which we cannot know), or do we act as “stewards” of their *capacity* for future welfare?

This ambiguity justifies a move away from “noisy” public deliberation toward the kind of dedicated role this paper formalizes below as an “Agent‐Specific Expert” (Section 3), who can leverage tools like superforecasting or neurobiological data to bound these future welfare states. Framing the problem as one of intergenerational externalities, DD as currently practiced can be seen as a “market failure” of representation—one that a structured Six‐Actor Framework is a candidate to help internalize, though presumably not the only one. (A related but distinct literature on formal impossibility results for preference aggregation—for example, Arrhenius [16] on population axiologies, echoing social‐choice impossibility theorems and Mookherjee [17]—shows that difficulties of this kind can arise even among fully capable participants; we set this literature aside here as a separate problem from voicelessness.) Taken together, the boundary problem, the JSP's reliance on a hypothetical rather than an actual consensus, and Gosseries's voiceless/toothless distinction all point to the same conclusion reached directly in Section 2.1: representing those who cannot deliberate requires a fundamentally different procedure from deliberation itself, one this paper develops as the Six‐Actor Framework in Section 3.

### 2.3 Empirical Evidence of Deliberative Failure

Empirical work from development economics and political economy complicates the assumption that more participatory mechanisms reliably improve outcomes for those they are meant to serve. In Uganda, Björkman and Svensson's [18] widely‐cited experiment found that community monitoring produced large gains in health outcomes—but a large‐scale replication roughly a decade later, using a similar design at greater scale, found no effect on utilization or health outcomes in any treatment arm [19]. A related follow‐up helps explain why: community monitoring paired with independently‐generated performance information sustained improvements over the long run, while monitoring alone, without that information, had no effect on quality of care or health outcomes [20]. In India, Banerjee et al. [21] found that giving village education committees information and encouragement to participate had essentially no effect on either participation or learning outcomes, while a targeted volunteer‐tutoring intervention run alongside it substantially improved test scores. In Indonesia, Olken [22] found that grassroots participation in monitoring local infrastructure spending—invitations to village accountability meetings, comment forms—had little effect on missing expenditures, while increasing the probability of a government audit sharply reduced it. And Giné et al. [23] find, in a randomized infrastructure experiment in India, that a data‐driven allocation mechanism outperformed a one‐person‐one‐vote referendum by 25%, with the referendum systematically favoring large landholders over the small and marginalized‐caste farmers for whom the infrastructure gains were largest. Across these settings, the common thread is not that participation is worthless, but that unstructured voice, unaccompanied by independent information or expertise, systematically underperforms—exactly the gap the Six‐Actor Framework's technical‐expert and agent‐specific‐expert roles are designed to fill.

Practical concerns also include power asymmetries. It is well known that individuals from marginalized groups are more often dismissed or talked over [24]. Kennedy et al. [25] demonstrated that even in online forums where sociodemographic characteristics were blinded, females, non‐whites, and the elderly contributed less. Individuals also often lack stable underlying preferences until they are queried, and struggle with affective forecasting, or predicting their own future wellbeing (Section 1). And where participants' goals are not fully aligned, there are strategic reasons, indeed often strong incentives, to misreport one's inputs [26]—a distinct problem from voicelessness, since it can arise even among fully capable, fully included participants, but one that further undercuts confidence in unstructured deliberation as a reliable aggregation mechanism.

Recent analysis by Das [27] regarding global health highlights a further tension. He argues that foreign aid can suffer from an “aid Dutch disease” allowing governments to bypass the local legitimacy needed to raise taxes. In health policy, popular wants and efficiency often clash; for instance populations may demand antibiotics where they are not medically efficient. Das warns that rushing to participatory action risks in practice cementing “elite cabals” that control the production of knowledge, suggesting that we need robust systems of expert adjudication rather than just more deliberation.

Taken together, this evidence suggests that the failure of participatory mechanisms in practice is not incidental but predictable: where voice is unaccompanied by information, expertise, or genuine accountability, it tends to reproduce rather than correct existing power asymmetries, for exactly the beneficiaries this paper is centrally concerned with. Section 3 turns to how a more structured allocation of roles might do better.

## 3 The Six‐Actor Framework: A Path Forward

The preceding analysis suggests that even if some version of full and equal democratic participation remains the ideal, it is in most cases practically infeasible to implement. This realization does not imply that the only alternative is a technocracy where experts dictate all policy. It does, however, imply that different agents will end up with different weights on their input and different mechanisms for incorporating that input, including indirect approaches such as explicit or implicit delegation via proxies. In this section, I outline a flexible structure for these possibilities, providing guidelines for which approaches are most defensible in specific circumstances.

### 3.1 A Taxonomy of Stakeholders and Experts

Standard policy debates often focus on a tension between elected officials and technical specialists against a mix of target populations and laypeople. To better explore the space of pertinent stakeholders, I propose a taxonomy of six relevant categories of decision‐makers:

1. Policymakers: Elected or appointed government representatives; major multilateral organizations such as the World Bank, UN, or WHO; and major philanthropists like the Gates Foundation or Coefficient Giving.
2. Technical Domain Experts: The group with the most scientific knowledge regarding the substantive topic, such as disease transmission, bioethics, or the relationship between regulation and economic growth.
3. Target Population: The group of people (or other relevant agents) most directly impacted by the policy decision.
4. General Laypeople: Contemporaneous society as a whole.
5. Benevolent Partisans: Those closest to potentially voiceless members of the target population, such as parents or caregivers of young children and the severely disabled, or those who work with animals.
6. Agent‐Specific Experts: Individuals with specialized knowledge regarding the patterns of views or welfare of the voiceless. This includes developmental psychologists for children, animal behaviorists and ethicists for nonhuman animals, and futurists,^5^ cultural historians, or institutional economists for potential generations.

This latter framework also encompasses researchers studying one's own sufficiently distant future self, which cannot literally participate in decisions that affect it any more than a future generation can [31], as well as evolutionary biologists and neuroscientists who interpret non‐verbal signals and brain data to complement standard communication methods.^6^ When outcomes are rare, uncertain, or transpire over time then it is especially important to listen to others with more experience (whether direct or research‐based), and indeed empirical evidence has shown that delegation is better in the context of intertemporal social decisions [32].

### 3.2 Expert Guidance, Delegation, and the Crowd

Once a stakeholder has been identified as relevant, a further question is how much weight to give their input relative to others', and through what mechanism. Every empirical or theoretical model for when people make predictable mistakes regarding their own preferences implies a model for how to overcome those mistakes. How to incorporate these views depends on the expertise behind the model. While the “wisdom of the crowds” performs remarkably well in many situations, it fails in others [33]. Researchers who study this phenomenon can help distinguish these situations and propose adjustments, such as eliciting second‐order beliefs [34].

Shared decision‐making is often the optimal approach, where experts (both domain and agent) work with the target population to tee up factors and pitfalls while leaving room for private preferences. Athey et al. [35] demonstrated that using an expert‐designed app for family planning worked better for multiple outcomes than letting agents choose entirely for themselves. If clinicians and patients cannot reach consensus, it is often preferable for the professional to take the lead [36]. Indeed, people often voluntarily delegate complex or technical decisions in their own lives—especially in the medical arena—by seeking recommendations from friends and family with more experience.

Furthermore, while procedural fairness often suggests focusing on those most directly involved, a pursuit of “moral realism” may require widening the scope to the general population (#4). This seeks to learn about the “true” normative parameter and avoid the self‐interested bias of a specific target group. For example, determining where “being dead” falls on a life satisfaction scale is a necessary input for policy trade‐offs; results from representative samples in Brazil, China, and the UK [37] are sufficient to conclude that it should be above zero but below the midpoint of the scale. Thus death is rated as a substantially bad outcome, but not the worst imaginable—corroborating the existence of “states worse than death” familiar from quality‐of‐life research, which provides clear empirical guidance for prioritizing extreme morbidity interventions.

Taken together, these considerations point to a single underlying question for each of the six actors: not simply whether their input is available, but whether it is the right kind of input for the decision at hand—crowd wisdom, domain expertise, agent‐specific interpretation, or some blend of these. Section 3.3 turns this question into a concrete default mapping.

### 3.3 Indirect Representation and the Surrogate Choice

Ideally, the voiceless would be represented by neutral, benevolent agents external to the decision itself—with no personal stake in the outcome—and with full information. Benevolent Partisans (#5) like parents are well‐placed regarding altruism and private information, though they are not entirely neutral and may miss predictable changes in outlook over time.^7^ Agent‐Specific Experts (#6) are an extension of this logic; they are more neutral and possess broader big‐picture knowledge, albeit with less individualized private information.

To help policymakers navigate these choices, the following matrix outlines a default mapping of roles for each surrogate based on the scale and nature of the decision:

**Table:** 

Decision Level	Recommended Surrogate	Primary Rationale
Individual/Personal	Benevolent Partisan	High private information; high affective proximity
Systemic/Legislative	Agent‐Specific Expert	Requires neutrality and large‐scale signal interpretation
Long‐Horizon/Global	Agent‐Specific Expert	Ability to forecast social change and multi‐generational values

Expert opinion is particularly necessary for the far future or animal welfare, where natural partisans are less prevalent than for children. Evolutionary biology allows us to understand the mapping from environment to preferences, enabling us to extrapolate welfare estimates to settings where unobservable outcomes cannot be directly ascertained. While experts may disagree and are subject to their own incentive alignment problems, any progress in utilizing this expertise is superior to the status quo where the voiceless are often not counted at all. Acknowledging these inherent limitations is a concrete first step toward crafting more ethically defensible policies.

In sum, the choice between Benevolent Partisans and Agent‐Specific Experts—and the weight given to each—should track the trade‐off the matrix above makes explicit: private information and affective proximity favor partisans, while neutrality and large‐scale forecasting favor experts. Section 4 applies this mapping to three concrete policy domains.

## 4 Illustrative Applications and Frameworks

The preceding sections establish the practical necessity of surrogacy for the voiceless and propose a Six‐Actor Framework to structure that representation. However, as the motivating example of trading consumption for health illustrates, the challenge is not merely one of inclusion but of informational fidelity. Even for enfranchised agents, we frequently rely on expert guidance to correct for predictable affective forecasting errors, such as the tendency to mispredict future well‐being following significant life changes, or assessing what health and economic scenarios are likely to actually exist in the medium‐ or long‐term future. For the voiceless, this “mechanical logic” of surrogacy is not an optional paternalistic addition but the primary engine of any ethically defensible policy.

The choice between a Benevolent Partisan (#5) and an Agent‐Specific Expert (#6) is not arbitrary: as the matrix in Section 3.3 makes explicit, it depends on the scale of the decision and the type of information required to represent the silent stakeholder. Partisans provide essential private information at the individual level, appropriate for personal and family‐level decisions; agent experts utilize neutral, big‐picture data to counter‐act partisan biases or the “presentist” preferences of current voters, appropriate as decisions scale toward the systemic or long‐horizon. Real‐world precedents for this stewardship model already exist at that systemic end: the UK All‐Party Parliamentary Group on Future Generations serves as a Category 6‐style body dedicated to ensuring that long‐term policy implications are explicitly considered in current legislation, and Wales' statutory Future Generations Commissioner performs a similar role with direct legal standing to comment on and challenge public‐body decisions. In more technical or systemic contexts, these agent‐experts may increasingly rely on tools like “superforecasters” or prediction markets to bound future perspectives and social value shifts with a level of rigor that raw deliberative sessions cannot achieve.

### 4.1 The Disvalue of Death by Age: Bioethical Metrics

The most prevalent international method for comparing health outcomes is the DALY (disability‐adjusted life‐year), which requires multiple explicit normative judgments regarding the badness of various morbidities compared to death. While we should place positive value on inputs from specific disease experts (#2) and the target population (#3), these groups necessarily have incomplete information and are likely to exhibit home biases, yielding a lack of biological and philosophical consistency.

A key input here is the relative value of deaths at different ages—a tension that became particularly salient during the Covid‐19 pandemic. One stark choice involves whether there is a socially‐relevant “discontinuity at birth”: should we expend significantly more resources to save a 1‐day‐old infant than a fetus 1 day before birth? Many Agent‐Specific Experts (#6)—including developmental psychologists and neuroscientists—suggest that no biological discontinuity exists in the capacity for future welfare.^8^ This claim is not directly testable by asking the fetus or infant, but it is at least indirectly corroborated: parental bereavement (#5) does not appear to track any sharp discontinuity at birth either, which is suggestive—not because a parent's grief is itself a measure of the infant's own welfare loss, but because bereavement intensity is a revealed belief about how much moral weight even the most affectively invested party assigns to the loss. Perhaps more surprisingly, a survey of general laypeople (#4) reveals a similar pattern [41], reinforcing that this is not an idiosyncratic feature of parental grief.

By re‐weighting DALY inputs using expert developmental analysis (#6) to estimate unobservable potential welfare directly, and treating the revealed beliefs of benevolent partisans (#5) and laypeople (#4) as corroborating rather than substituting evidence, policymakers can move toward a more consistent valuation of life near birth. This structured approach, an appropriate blend of expert and other opinions, would noticeably shift resource allocation toward late‐term stillbirth prevention.

### 4.2 Animal Welfare: Beyond Anthropomorphic Optics

Public referendums on animal welfare, such as those concerning battery cage bans, are frequently driven by the “anthropomorphic optics” of General Laypeople (#4). Voters typically ask what *looks* distressing to a human eye rather than what is actually welfare‐diminishing for the animal. This creates a market failure in welfare preferences where “marketable” standards (like “cage‐free” labels) are prioritized over biologically effective ones.

The framework corrects this by elevating Agent‐Specific Experts (#6), such as ethologists and animal behaviorists, who interpret stress signals like cortisol levels or stereotypies that remain unobservable to the layperson. This is particularly critical for species that lack natural human advocates; while domestic animals have many partisans (#5), the welfare of trillions of nonhuman animals—including the billions of currently existing carp worldwide who can live for decades—is often entirely ignored.

Applying this structured input would change the policy decision from “optics‐based” bans to biologically grounded standards. For example, behaviorists might demonstrate that complex environmental enrichment is more vital for a species' flourishing than the square footage of a cage—a nuance that a standard deliberative session among humans would almost certainly miss. Evolutionary biologists (#6) further assist by extrapolating welfare needs for species where direct preference elicitation is impossible, ensuring that policy is not limited to “charismatic megafauna” but extends to all sentient agents.

### 4.3 Population Ethics and Global Health Priorities

Global health policymakers currently face a stark inconsistency: we count the lives of non‐existent future children when valuing malaria bednet programs, yet we discount them entirely when valuing family planning interventions.^9^ Bednet programs are often viewed through the lens of total utilitarianism, counting everyone equally, while family planning often leans on person‐affecting views, where a potential child doesn't “count” because they aren't a “they” yet.

Scientific technocrats (#2) are not well‐placed to adjudicate these deep philosophical disagreements (indeed the two relevant groups appear to disagree in this case), nor can we easily “follow the public” (#3 and #4) when the beneficiaries in question are hypothetical for now. Instead, we need an amalgam of inputs:

- Benevolent Partisans (#5): Parents may offer some insight into the context a potential child would be born into, though this is not a proxy for the child's own welfare. Separately, and in their own right as a directly affected party (#3) rather than as a stand‐in for the potential child, parents' reproductive autonomy—including the specific context of “unwanted” pregnancies—is a legitimate interest independent of the population‐ethics question addressed here.
- Agent‐Specific Experts (#6): Applied ethicists to chart a path through unsettled population axiology, and futurists or institutional economists to model the welfare of “necessary” future people (those who will exist regardless of our policy choice).

By using Category 6 experts to translate these abstract ethical tensions into concrete “moral weights” (a process recently explored by organizations like GiveWell^10^), policymakers can develop a consistent framework for global priorities along these lines. This structured consideration takes the goal of inclusive representation more seriously than methods that, in practice, exclude the very populations they intend to serve.

Across all three domains, the same pattern recurs: voicelessness is not a peripheral complication to be noted and set aside, but the central design constraint the policy process must be built around—which is precisely what the Six‐Actor Framework is built to make tractable, rather than merely acknowledged.

## 5 Conclusion

This article has argued that while the impetus for incorporating public input into policy decisions—particularly in health, biotechnology, and environmental policy—is well‐founded in principles of fairness and information aggregation, standard deliberative democracy faces a structural barrier. Crucially, key stakeholders—young children, nonhuman animals, future generations, and individuals with severe cognitive impairments—suffer from a practical incapacity for reasoned engagement. Because these voiceless groups often constitute the majority of affected beneficiaries in high‐stakes domains, relying solely on articulate participants introduces a systematic selection bias. Procedurally, direct deliberation with silent constituencies is impossible; some form of proxy representation, stewardship, or signal interpretation is functionally unavoidable.

Rather than treating surrogacy as an unprincipled fallback, this paper's central contribution is to operationalize it through a structured Six‐Actor Framework. By distinguishing between Benevolent Partisans (#5), who offer essential private information and affective proximity at the individual level, and Agent‐Specific Experts (#6), who provide neutral, large‐scale signal interpretation and long‐horizon forecasting, the framework provides a pragmatic architecture for policy design. Explicitly structuring proxy input is not a concession that undermines democratic ideals; rather, it is a more faithful realization of the All‐Affected Principle than procedures that default to excluding silent populations altogether.

The illustrative applications in this paper demonstrate how this framework resolves concrete bioethical and economic trade‐offs—from re‐weighting DALY inputs near birth, to replacing anthropomorphic optics in animal welfare with ethological standards, to navigating a path between person‐affecting and total utilitarian perspectives in population ethics. Extending this logic reveals a further, more literal parallel: temporal incapacity is not limited to generations that do not yet exist, but plausibly extends to an enfranchised adult's own sufficiently distant future self, who is no more able to participate in today's decision than a not‐yet‐existing person is. This is a stronger claim than the familiar observation that people are simply bad at predicting their own future preferences: Jamison and Wegener [46] find that people represent their distant future selves much as they represent other people entirely, rather than as a continuous extension of the present self—suggesting a genuine discontinuity in the decision‐making subject, not merely an error correctable by better information. If this is right, the same logic that justifies Benevolent Partisans and Agent‐Specific Experts for the voiceless applies with comparable force to a person's own future self: not as a debiasing exercise, but as a second, more familiar instance of the very structural problem this paper has addressed throughout.

Looking ahead, significant work remains in refining the methodologies of “agent expertise.” Future research should focus on enhancing the empirical validity of these proxies—specifically in decoding non‐verbal welfare signals, forecasting long‐term value shifts, and refining superforecasting tools. Applying this structured approach to emerging bioethical frontiers, such as the moral status of digital minds or human genetic enhancement, will be critical. Ultimately, unstructured deliberation in health and science policy is often practically infeasible and epistemically unreliable. Embracing a structured, ethically explicit framework allows global priorities research to focus where it can achieve the greatest positive impact across billions of current lives, future populations,^11^ and trillions of nonhuman animals.

## Funding

The author has nothing to report.

## Conflicts of Interest

The author declares no conflicts of interest.

## Data Availability

The author has nothing to report.

## Endnotes

1 See Wilson & Gilbert [1] for the canonical general reference here, and Halpern & Arnold [2] for an application to medicine and in particular clinical decision‐making.

2 Drawing heavily from Cohen [6]. The epistemic rationale is linked to Elster and others, while the process rationale is linked to Habermas and others.

3 Perhaps counterintuitively, the most ‘privileged’ members of society, precisely because they do not need to advocate for their own interests, may be best placed to neutrally advocate for the voiceless.

4 For instance, we find “Rather than imagine a (hypothetical and nonhistorical) direct agreement between all generations, the parties can be required to agree to a savings principle” in Rawls [13].

5 This covers a broad range of possibilities, from general practice for thinking more long‐term [28, 29] to especially around specific policy design choices that impact and improve future generations [30].

6 And of course computer scientists if the relevant group is digital minds or sentient AI.

7 See Ypi [38] for a previous defense of one instantiation of partisanship in the political context, and Nelson et al. [39] for discussion of empirical and theoretical conflicts of interest imparted by ‘experience’ in the context of parents and more broadly.

8 The Global Burden of Disease project [40] suggested one possible conceptual method for both incorporating stillbirths and potentially devaluing infant deaths, showing how much this would change the relative importance of various common global interventions.

9 See for instance the recent analysis by GiveWell [42] section 2.2, or a modest indirect loss in UK government guidelines [43] section 4.2.5. Luyten, Verbeke & Schokkaert [44] review the academic literature.

10 For instance the approach to ‘moral weights’ described by GiveWell [45] started with internal ‘experts’ only, added direct beneficiary preference elicitation, and then backed away from simply using those raw results.

11 In particular, ‘longtermism’ is articulated and defended in Greaves & MacAskill [47], with a nuanced response in Persson & Savulescu [48].
